# A novel diabetic retinopathy detection from fundus images using hybrid quantum convolutional neural network models

**DOI:** 10.1038/s41598-026-49227-2

**Published:** 2026-05-05

**Authors:** S. R. Menaka, Suresh Muthusamy, Prabhjot Kaur Sidhu, Abhinandan Routray, G. Uma Maheswari, Nebojsa Bacanin

**Affiliations:** 1 Department of Information Technology, K.S.R. College of Engineering (Autonomous), Tiruchengode, Namakkal, Tamil Nadu India; 2 Department of Electrical and Electronics Engineering, Kongu Engineering College (Autonomous), Perundurai, Erode, Tamil Nadu India; 3 Department of Information Technology, Maharaja Surajmal Institute of Technology, Affiliated to GGSIP University, Janakpuri, New Delhi India; 4 Manipal Institute of Technology, Manipal Academy of Higher Education, Manipal, India; 5 Department of Computer Science and Engineering, RMK College of Engineering and Technology, Puduvoyal, Thiruvallur, Tamil Nadu India; 6 Faculty of Informatics and Computing, Singidunum University, Danijelova 32, Belgrade 11000, Serbia, Danijelova 32, Belgrade 11000 Serbia; 7 Department of Mathematics, Saveetha School of Engineering, SIMATS Thandalam, Tamilnadu 602105 Chennai, India

**Keywords:** Diabetic retinopathy, Fundus images, Quantum computing, CNN, Retina, Image processing, Computational biology and bioinformatics, Diseases, Engineering, Health care, Mathematics and computing, Medical research

## Abstract

Diabetic retinopathy (DR) diagnosis from digital fundus images is a long-standing topic of research in medical image processing. The determination of optic disk boundaries in two-dimensional retinal images is difficult due to blurred edges, which makes this field in need of improvement. All these problems cannot be solved by a single technique. An efficient algorithm for identifying DR-related retinal changes and structure is still needed. If DR is recognized and treated in a timely manner, visual deterioration can be managed or avoided. It is based on telemedicine analysis of color fundus pictures or clinical evaluations by medical doctors. However, due to intrinsic human subjectivity, both systems are time-consuming, labor-intensive, and prone to inaccuracy. Due to their great specificity and sensitivity, automated methods capable of analyzing color fundus pictures have become important for the general deployment of DR screening. To study the existence of DR-related characteristics and to cope with the various diabetes severity diagnosis phases, a hybrid quantum convolutional neural network (HQCNN) is presented. Kaggle fundus images database is utilized to test and train the network. Finally, the presented work is compared for analyzing efficiency using the system of measurement like precision, specificity, accuracy, sensitivity, and f1 score. The proposed work obtains accuracy of 98.89%, sensitivity of 99.37%, specificity of 99.57%, precision of 98.89%, and F1 score of 97.58%.

## Introduction

Diabetic retinopathy (DR) is the main reason for sight loss in individuals of employed people. Diabetes mellitus affects around 420 million individuals globally. This disease’s occurrence has more than twice in the last 30 years^1^ and is only likely to rise more, especially in Asia. One-third of diabetics are projected to be recognized with DR, a serious eye illness that can lead to permanent sight loss. Diabetic will emerge as the most dangerous and seventh foremost reason of world mortality rate by 2030, as per to the World Health Organization^2^. DR develops because of severe diabetes damaging microscopic blood vessels within the retina.

Considering DR is a chronic illness whose seriousness is determined by the quantity and kind of injuries in the fundus picture, early identification and treatment are critical. It also enhanced the effectiveness of DR scaling systems, which were tested with great number of high-resolution pictures captured in a variety of settings, as opposed to conventional hand-designed feature-based techniques^3^. Various photograph categorization methods based on characteristics have been developed over the years to identify the DR seriousness level fundus photos. Usual fundus checks can actually reduce DR-related vision loss. Veins, optic discs, and the macula are the key elements of a functioning retina, and any abnormalities in these elements are indicators of eye illness^4^.

Early identification, that is crucial for a favorable prognosis, requires experts and labor are time-consuming. Thedilemma in locations where trained medical infrastructures are generally scarce. Furthermore, the laborious aspect of DR screening procedures encourages reader inconsistencies. Lastly, given the global rise in the incidence of diabetics and its related visual problems, manual techniques of detection may find it difficult to keep up with the increasing desire for monitoring facilities^5^. Automated strategies for diagnosing DR are critical to resolving these issues.

Quantum computing (QC) is such successful topic that may be able to assist in addressing this issue with completely new structures. More study has recently been performed to reduce the computing cost of train big systems using learning approaches wherein frameworks are generated with pre-trained characteristics on big databases and just the last layers of the system are trained up with fresh input for the desired tasks. To reach much superior efficiency, researchers investigated and documented the viability of integrating deep learning (DL) architectures into the quantum domain^6^. Simultaneous to latest events in conventional Machine Learning (ML), intellectual curiosity in quantum ML has increased dramatically^7–9^. Parameterized quantum circuits (PQCs) are a QC concept having powerful parallelization with descriptive features. With PQC, several scientists have built quantum ML systems. Schuld et al.^10^ utilised PQC to build a circuit-centric quantum classification model in 2020. The experiment using the database revealed that the quantum classification performed well. Zeng et al.^11^ suggested a fusion quantum model of NN with a ladder circuit layout and a comprehensive assessment technique in 2022. Under all binary and multi-classification tests, the system exhibited consistent and good accuracy. Peruzzo et al.^12^ developed and achieved a Variational Quantum Eigen solution (VQE) combining a photon quantum workstation and a conventional processor in 2014.McClean et al.^13^ updated the optimizing technique of the variational quantum method in 2016. Several quantum ML algorithms depending on PQC have been presented when PQC was initially introduced^14–20^. There has been proven the quantum ML system centred on PQC outperforms the traditional technique in terms of animated efficiency and computing capability^21,22^.

We develop a composite quantum-classical framework for DR categorization by integrating PQC with a conventional NN. The hybrid version not only makes use of PQC’s superior performance and also retains the properties of a traditional NN. Quantum convolution kernels produced by PQC make up the quantum convolutional layer. This framework is referred to as a HQCNN in this paper; simulation studies reveal that HQCNN has excellent learning capacity and good picture categorization reliability. The choice to use a Quantum Convolutional Neural Network (QCNN) for addressing the problem of diabetic retinopathy detection would be influenced by several factors that align with the unique properties and capabilities of quantum computing. Potential Quantum Advantage: Quantum computers have the potential to solve certain problems faster or more efficiently than classical computers due to their ability to perform certain calculations in parallel and exploit quantum interference. For tasks involving large amounts of data and complex computations, such as medical image analysis, there’s a possibility that quantum algorithms could provide advantages over classical methods.

Inherent parallelism: Quantum computers can process information in parallel using quantum superposition. This property could be particularly advantageous for image-based tasks like diabetic retinopathy detection, where convolutional operations are used to extract features from images. Quantum parallelism might speed up the processing of large image datasets.

Feature extraction: Quantum computers can process and extract features from data in ways that differ from classical approaches. Quantum gates and circuits can perform transformations that might capture intricate features within medical images more effectively than classical convolutional layers.

Quantum data representation: Quantum machine learning allows for the encoding of data using quantum states, which can potentially lead to more compact and efficient representations of complex datasets. In the context of diabetic retinopathy detection, this could help in reducing the computational requirements for processing large medical image datasets.

Higher-dimensional data: Medical images are often high-dimensional data, and quantum computers have the potential to handle higher-dimensional data more efficiently due to their inherent nature of working with quantum states.

Potential for Enhanced Sensitivity: Quantum algorithms might be able to detect subtle patterns and correlations within medical images that are challenging for classical algorithms to uncover. This could be crucial for accurately detecting early stages of diabetic retinopathy.

Exploring quantum neural architectures: Researching and applying quantum neural network architectures like QCNNs to real-world problems like diabetic retinopathy detection could provide valuable insights into the capabilities and limitations of quantum machine learning techniques. This study’s primary participation is,

- To avoid generalization and overfitting problems a larger dataset is utilized for the offered model.
- To prevent diabetes complications, the HQCNN model is offered here to diagnose the problem earlier.
- Results are analyzed for the proposed work with other state-of-the-art algorithms to evaluate the performance of the proposed work.

The remaining work is systematized as follows. “Literature survey” delivers a review of the literature concerning DR detection utilizing diverse methods including quantum computing-based assessment. “Methodology” describes the planned work approach. “Results and discussion” includes a quality assessment that validates the effectiveness of the projected work. “Conclusion” provides our planned work summary.

## Literature survey

Hossain et al.^23^ developed an efficient Variational QC based technique for detecting the presence of malaria inside a Red Blood Cell (RBC) picture by classifying an optimal characteristic set taken from a series of RBC pictures. The characteristic collection is optimized using minimum Redundancy Maximum Relevance (mRMR) and Principal Component Analysis (PCA). Following determining the existence of malaria by VQC, they implemented a rule-based intelligent model to recognize the different forms of malaria.

Houssein et al.^24^ suggested a novel Hybrid Quantum-Classical CNN (HQ-CNN) system for predicting COVID-19 using chest radiography pictures. To calculate convolution operations on a quantum gadget, the HQ-CNN framework uses Random QC (RQC) as a quantum convolution layer. The suggested approach may differentiate COVID19, viral pneumonia, and bacterial pneumonia from normal patients.

Nandy et al.^25^ suggested a QC application in Siamese design and investigated its performance in exudate impacted retinal photo patch recovery. QC in Siamese system design might be useful for picture patch feature contrast and retrieving operations. Even though maintaining high-dimensional interior product field is a limitation, the circuit with a minimal selection of qubit depicts exudate-impacted retinal picture spots and gets comparable spots from the patch repository.

Tamilvizhi et al.^26^ introduced a Quantum Behaved Particle Swarm Optimization centered Deep Transfer Learning (QBPSO-DTL) framework with good accuracy for sugarcane leaves illness identification and categorization. To find the impacted areas there in leaf picture, the suggested QBPSO-DTL approach includes the creation of appropriate area expanding segmentation. Furthermore, the SqueezeNet network is used as a key point harvester, and the Deep Stacked Auto Encoder (DSAE) framework is used as a classifier. Lastly, the QBPSO method is used to tune the DSAE framework’s hyper-parameters.

Depending on multifractal geometries, Abdelsalam and Zahran^27^ recommended a unique procedure for timely DR identification. Image investigation of macular Optical Coherence Tomography Angiography (OCTA) with first non-proliferative DR diagnosis (NPDR). Applying a supervised ML technology such as the Support Vector Machine (SVM) method for automating the diagnostic procedure as well as increasing the correctness.

Gundluru et al.^28^ suggested a technique for picking the desirable attributes using PCA. The suggested model’s database is derived from the publicly available UCI ML repository that includes repetitive and superfluous characteristics in its natural form. The method of selecting and extracting the relevant characteristics from the database was surpassed by the Harris hawks optimization technique.

Jadhav et al.^29^ proposed a unique method for establishing automatic DR diagnosis by assessing retina defects, for instance harsh exudates, hemorrhages, microaneurysms, and gentle exudates. DR analysis phases in this case were pre-processing, optic disc elimination, vein withdrawal, abnormality segmentation, and categorization. Furthermore, the Deep Belief Network (DBN) centered classifying scheme was applied to classify photos into 4 groups: regular, mild, medium, and serious. The updated approach entitled Modified Gear and Steering centered Rider Optimization Algorithm (MGS-ROA) were utilized to perform the best characteristic picking and weight updating in DBN.

Ayala et al.^30^ used a CNN to analyze a fundus picture to discriminate against the ocular anatomy and sense DR. Transfer-learning process is used to optimize the model’s settings for matching a picture with the relevant label. Regarding labels, the setup is educated and verified by a collection of clinical fundus oculi pictures and a pathological seriousness scale identified in eyeball. The seriousness scale categorizes the photographs into 5 groups, ranging from a normal eyeball to the existence of proliferative DR.

Menaouer et al.^31^ suggested a mixed DL strategy to DR identification and classification based on the retinal hazard associated with the severity of visual ischemia utilizing the deep CNN technique and two Visual Geometry Group (VGG) system designs (VGG16 and VGG19). Indeed, the classification of DR is concerned with comprehending the pictures and its contexts in relation to the classes. This mixed technique is totally automated from start to finish, eliminating the requirement for human extraction of features.

This article has reviewed the most recent automated systems of diabetic retinopathy detection and classification that used deep learning techniques. Also, quantum-based research works are also reviewed in this section. Based on the above literature study, it is identified that most of the DR detection mechanisms are used deep learning framework, but they require more time for training. Deep learning models made on quantum computers are more powerful and faster compared to the models that are run on classical computers. Quantum deep learning helps to solve problems with complex correlations between inputs that are challenging to solve with traditional classic computers.

## Methodology

**Fig. 1:**
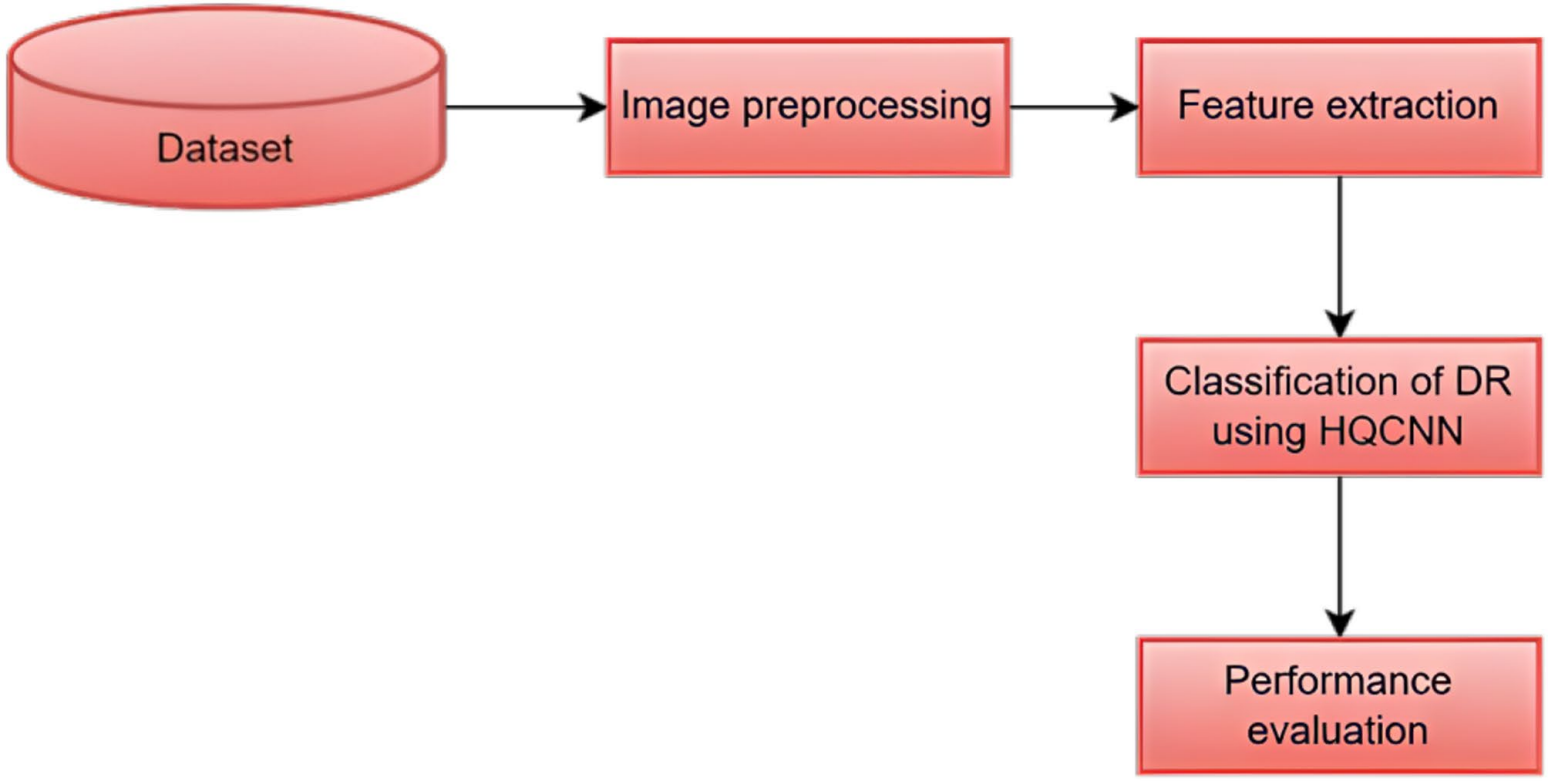
Flow of the suggested work.

The methods of DR detection were described in this section of the article, together with detail concerning the database and the techniques used. The stream of the suggested work is represented in Fig. 1. proposed methodology consists of data acquisition, image preprocessing to improve image quality. Then feature extraction will be done and finally classification done using hybrid Convolutional Neural Network.

### Dataset

The Kaggle database, that is a systematized data source of fundoscopic images given by EyePACS, is used for DR identification. This EyePACS set contains 35,126 high-quality photographs varying in quality between $\:433\times\:289$ to $\:5184\times\:3456$pixels and captured with different cameras. Each picture is separated into 5 DR stages according on the type of the DR chronicity. Table 1 depicts the significant class imbalance between normal class and PDR class. To generalize the robustness toward major classes, several strategic procedures were implemented. First, Data augmentation techniques such as rotation, flipping and translation were applied to all classes during training process to increase the feature representation of minority classes and reducing bias in the majority classes. Second, Weighted Cross Entropy function was used which assign higher weight for misclassification of PDR and severe stages.

Further, stratified splitting was used to conserve class proportion across training, validation and testing set to ensure unbiased performance evaluation. To confirm that the “high accuracy” is not distorted by the majority class, including a Normalized Confusion Matrix and Class-wise F1-Scores. In addition to Kaggle dataset, test was conducted for both IDRiD and MESSIDOR data sets, consisting of 516 retinal fundus images for IDRiD and 1200 for MESSIDOR. These data sets were used for measuring the performance consistency of HQCNN.

**Table 1:** Distribution of DR classes across dataset spilt.

Distribution of images into DR classes	Class 0 (normal)	Class 1 (mild)	Class 2 (moderate)	Class 3 (severe)	Class 4 (PDR)	Entire
Total pictures	25,810	2443	5292	873	708	35,126
Training set	16,536	1563	3359	558	453	22,469
Validation set	5155	489	1088	175	142	7049
Testing set	4119	391	845	140	113	5608

### Image preprocessing

Preprocessing removes noise or fluctuation from the retinal fundus images and improves picture features and clarity. To eliminate artifacts and improve method efficiency, the preprocessing phase may also include noise removal and image improvement. Further classification of fundus images involves identifying, extracting, and dividing DED traits.

### Data augmentation

The incoming photos were resized to $\:224\times\:224$ before being utilized for network training. For efficient neural net training, a large dataset is necessary. Whenever trained on a small database, deep networks cannot generalize, leading in poor testing efficiency. Data augmentation, that utilizes and improves the present dataset fast and efficiently, is one remedy for this issue. The size of the training database affects the performance of DL algorithms. A larger dataset for sophisticated network design is required to avoid generalizing and overfitting concerns. The size of the medical images collection is usually rather small. A variety of data augmentation elements, which include the twisting, moving, spinning, and cropping fundus images, are utilized to solve this.

**Fig. 2:**
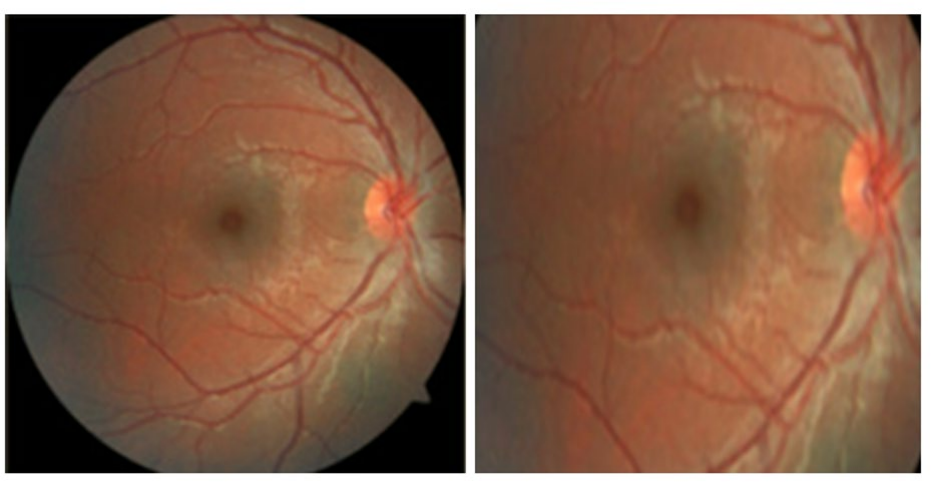
(**a**) Original image (**b**) Cropped image.

The retinal pictures are first cut from the middle and the borders. They modified the rotational degree $\:{(0-90)}^{^\circ\:}$ and travelled inside a specific reference frame to eliminate performing needless regions. Lastly, flipping is utilized to improve the results. Figure 2 displays the (a) actual image and the (b) cropped image. The cropping image will remove the dissimilar borders and improve the retinal structures such as optic disc and vessels. The settings and values utilized for data augmentation are exposed in Table 2.

**Table 2:** Data augmentation parameters and values.

Parameter	Value
Height shift	0.2
Width shift	0.2
Rescale	1/255
Rotation	90
Zoom	0.2
Shear	0.2
Vertical flip	True
Horizontal flip	True

### Image smoothing

To enhance feature extraction from all images in this approach, smoothing is utilized in the fundus images to eliminate minor features. Such a transformation (*T*) could be used to generate the smooth picture *S (a*,* b)* for any picture, *I (a*,* b).*

1 $$\:S(a,b)\:=\:T\:\left[I\right(a,b\left)\right]$$

We chose the median filter amongst different transformation algorithms based upon two quantified measurements: MSE and PSNR. Thus,

2 $$\:MSE=\frac{1}{ab}\sum\:_{i,j=1}^{a,b}[I\left(i,j\right)-S{(i,j)}^{2}$$

3 $$\:PSNR=10{log}_{10}\frac{256-1}{MSE}$$

Furthermore, median filter reserves corners for enhanced feature retrieval via contour let transformation. The median filter may be outlined for any window $\:W$as below:

4 $$\:S(g,h)\:=\:median\left\{I\right(g\:+\:k,h\:+\:l\left)\right\};\:(k,\:l)\:\in\:W\}$$

Herethemidpoint of *W* (*k*, l) ∈ Z^2^, and *T* ← median.

### Preliminaries

Computation of quantum is substantially separate from conventional computing that uses binary circuits since it is based on quantum mechanics. It possesses several key features, including superposition, entanglement, and unitary transformations, demonstrating its strong computing capabilities. We begin with the fundamentals of Qubits, Quantum operations, Entanglement and Measurement.

### Qubits

Likewise, to a binary bit in conventional calculation, a qubit is the fundamental unit of quantum computation. Qubits contain two fundamental states |0> & |1>, which correspond to the ground and excited stages of a 2-level quantum structure. Qubits can be in any superposition phase between |0> and |1>, unlike traditional bits, which are limited to holding a single result at a time:

5 $$\left| \psi \right\rangle = \alpha \:\left| 0 \right\rangle + \beta \:\left| 1 \right\rangle$$

Here α,β∈C denote likelihood amplitudes and fulfill |α|^2^+|β|^2^=1. Select {|0>, |1>} as an origin, then a few solo qubit state |ψ> was denoted through a difficult vector:

6 $$\left| \psi \right\rangle = [\alpha \:\beta \:| \in \:{C^2}$$

The term sealed quantum structure refers to a quantum structure that has no contact with the environment around it. Considering n closed qubits, which quantum modes are represented by as |ψ^1^>,⋯,|ψ^n^>. A composite framework built of such n qubits has a quantum mode |Ψ> = |ψ^1^> ⊗⋯⊗ |ψ^n^>, that is represented as |ψ^1^⋯ψ^n^> for accessibility. This linear space describes the linearity of an arbitrary *n*-qubit quantum phase: {|00⋯0>, |00⋯1>, |11⋯1>}. Several quantum states |Ψ> will be represented as superposition states in this linear space:

7 $$\left| \psi \right\rangle = \sum {_{i = 0}^{{2^n} - 1}} \alpha {\:_i}\left| \psi \right\rangle ,\alpha {\:_i} \in \:C$$

where |i> refers to the quantum phase specified through the binary way of i, for instance, |7> = |111>.

### Entanglement

Entanglement is the quantum phenomenon, used for correlating one state with the other. That is outcome of one qubit will determine the state of other qubit. If qubits are entangled, the combined quantum system’s status cannot be expressed as the tensor products of every individual qubit state, like the Bell state: $\left| {{\varphi ^ + }} \right\rangle = \frac{{\left| {00} \right\rangle + \left| {11} \right\rangle }}{{\surd 2}}$. It’s mainly used for identifying missed pathologies and also provides better accuracy for early stage disease diagnosis.

### Quantum gates

Qubit states can be controlled by quantum gates in QC. Unitary transformation U, defining UU† = I, defines the growth of a locked environment in quantum mechanics. For a quantum environment with the starting state$\left| {{\psi _0}} \right\rangle = \sum {_{i = 0}^{{2^n} - 1}} {\alpha _i}\left| i \right\rangle = {\alpha _i}$, a quantum gate executing a unitary transformation U acts like matrix-vector multiplication.

8 $$U\left| {{\psi _0}} \right\rangle = U\sum {_{i = 0}^{{2^n} - 1}} {\alpha _i}\left| i \right\rangle = {\alpha _i}\sum {_{i = 0}^{{2^n} - 1}} {\alpha _i}\left| i \right\rangle = {\beta _i}\left| i \right\rangle$$

### Quantum measurement

The data in the quantum environment is not directly approachable, and we must undertake a quantum measure to acquire it. A geometric experiment with W noticeable on qubit with level |ϕ> = *α*|0> + *β*|1⟩ yields 1 and − 1 through likelihood *p*(1) = |*α*|^2^ and *p*(− 1) = |*β*|^2^, accordingly. Consequently, intermediately shifts the quantum state to |0> or |1> following the observation. The outcomes of measurements are stochastic, and each observation can only provide one potential value with accompanying likelihood. As a result, we must redo the tests in order to obtain the most exact data regarding the state. The expected range of a given test observable *W* on state |*ϕ*⟩ is represented as follows:

9 $${\left\langle {\rm{W}} \right\rangle _{\left| \phi \right\rangle }} \equiv \left\langle {\phi \:\left| {\rm{W}} \right|\phi } \right\rangle = \left. {{{\left| \alpha \right|}^2} - {{\left| \beta \right|}^2}} \right|$$

$${\rm{Here}}\:W \equiv \:\left[ {\begin{array}{*{20}{c}}1&0\\0&{ - 1}\end{array}} \right]\:,\left\langle \phi \right| = {(\left| \phi \right\rangle )^\dag },\:{\rm{and }}\left\langle W \right\rangle \in \:[ - {\rm{1,1}}].$$.

### Quantum CNN based DR detection

Regarding data processing, QC depends on quantum mechanics postulates and properties (such as quantum bits, interfering, superposition, and entanglement). QC enables us to tackle complicated issues more efficiently and quickly than traditional computers (Aimeur et al., 2006). Qubits in quantum computers are like bits in conventional computers, a tiny unit of data processing. There are three possible states for a qubit: one, zero, or each at the same moment, a phenomenon referred to as linear superposition. In Hilbert space, a qubit is a state vector (Dunjko et al., 2016).

10 $$\psi = \left( {\begin{array}{*{20}{c}}\theta \\\delta \end{array}} \right) = \theta \left| 0 \right\rangle + \delta \left| 1 \right\rangle$$

### Parameterized QC

**Fig. 3:**
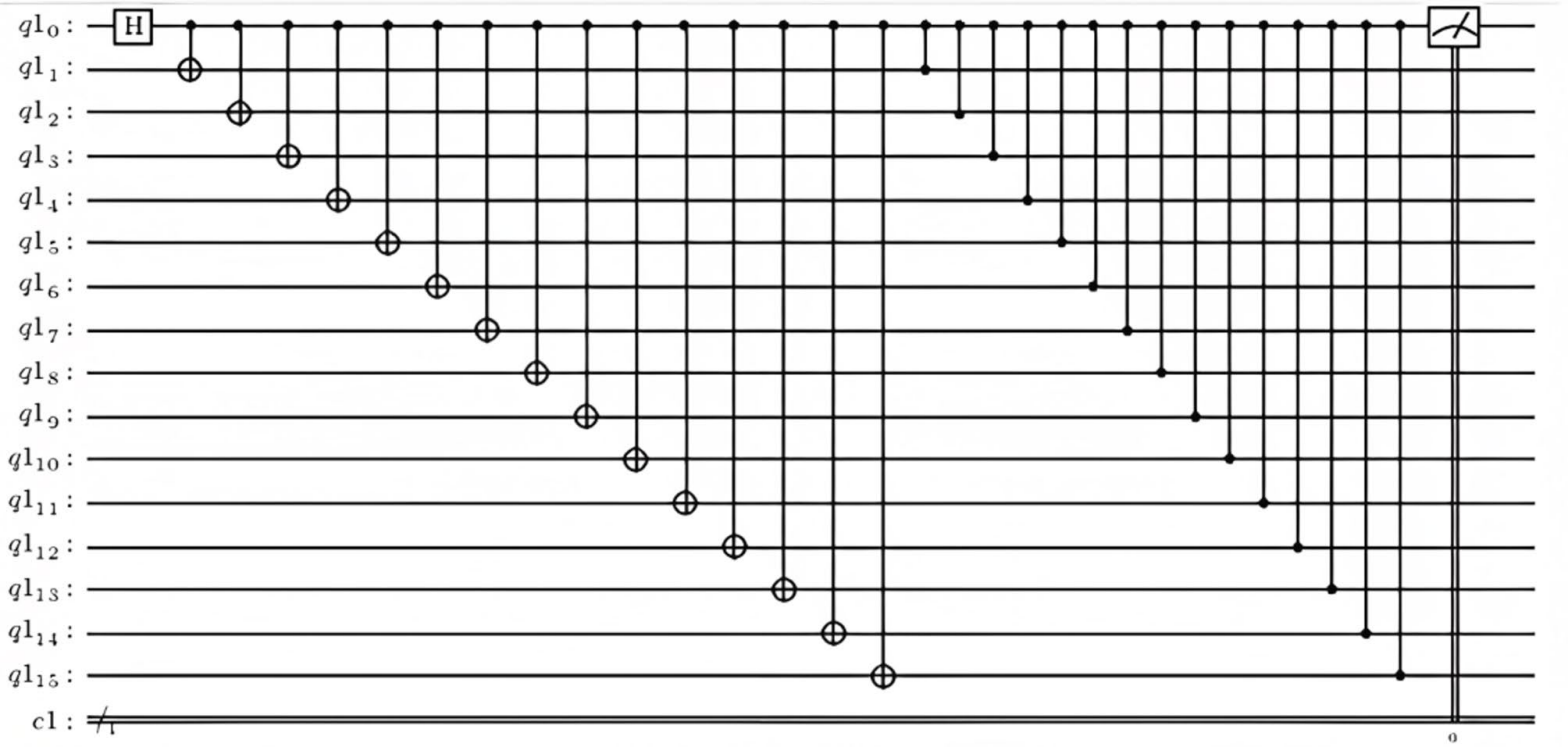
Parameterized quantum circuit diagram.

Parameterized QCs (PQCs) created with quantum gates are used to model quantum NN. PQCs can be highly efficient at yielding considerable inference at times. The traditional data is first processed and supplied into the PQCs. The traditional processor also monitors the readings from the model result at the output terminal. Thus, quantum classical frameworks are described in this way. This is done by loading quantum information from tensors into the TFQ. Cirq items are changed into string tensors using the tfq.convert to tensor method. SymPy entities are used to characterise the cirq.Circuit objects. TFQ operations are used to transform these tensors to traditional data. PQC-format quantum states are generated, and observations are collected. The predictions are made based on measured data. Cirq circuits are transformed to TensorFlow QC having specified information and readout qubits in this study. A model circuit is constructed using the Circuit Layer Builder module. A TFQ-Keras structure surrounds the model circuit in PQC. Two qubit gates link each input qubit to its readout and make up a QC. The parameterized quantum circuit is shown in Fig. 3. Each gate refers the unitary transformation which will be applied to qubits that will enable the encoding of classical image into quantum states for feature extraction.

### Quantum state preparation

When the quantum network is restricted to a certain amount of qubits and the quantum platform is employed to tackle classical difficulties, traditional data dimension compression is necessary.

While the quantum circuit needs reduced input dimensions, clinical significance is maintained during multistage compression process that preserves the features of retinal fundus images before moving on encoding.

Image refinement: The initial image is resized to 224 × 224 for pre-processing and augmentation to improve the image features such as optic disc and vessels.

Dimensionality reduction: The preprocessed image is down sampled to m × m size, where m = 4(4 × 4) to ensure feasibility under quantum simulation constraints.

Quantum mapping: The 16 pixels of downsampled 4 × 4 image are mapped to a total of m^2^ = 16 qubits for quantum encoding. This provides one-one mapping for the 16 pixels to 16 qubits.

Angle encoding: The number of pixels is scaled to [0, 1], and the picture is flattened to 1 × *m*^2^ vector, where *x* = [*x*_1_, *x*_2_. . *x*_m2_ ] and transform the vector to α angle data, using

11 $$\alpha = \pi x$$

Here *α =* [*α*_1_, *α*_2_ *. . α*_*m2*_ *]*. For an m2-input quantum framework, to encode a quantum state $\:{{\upphi\:}}_{\mathrm{i}\mathrm{m}\mathrm{g}}$, the angle data is used to compute the rotating angle of the rotating gate *R*_y_.

12 $$\left| {{\phi _{img}}} \right\rangle = \otimes \:\begin{array}{*{20}{c}}{16}\\{i = 1}\end{array}{R_y}\left( {{\alpha _i}} \right)\left| 0 \right\rangle$$

The entire test was done using a quantum simulator (pennyLane integrated with Tensorflow). While angle encoding is used for the current 1:1 mapping in the 16-qubit simulation environment, the amplitude encoding can be used for achieving higher resolutions images, where N pixels represented by *n = log*_2_*N* qubits, it will considerably reduce the hardware overhead for clinical datasets.

### Quantum convolutional layer

**Fig. 4:**
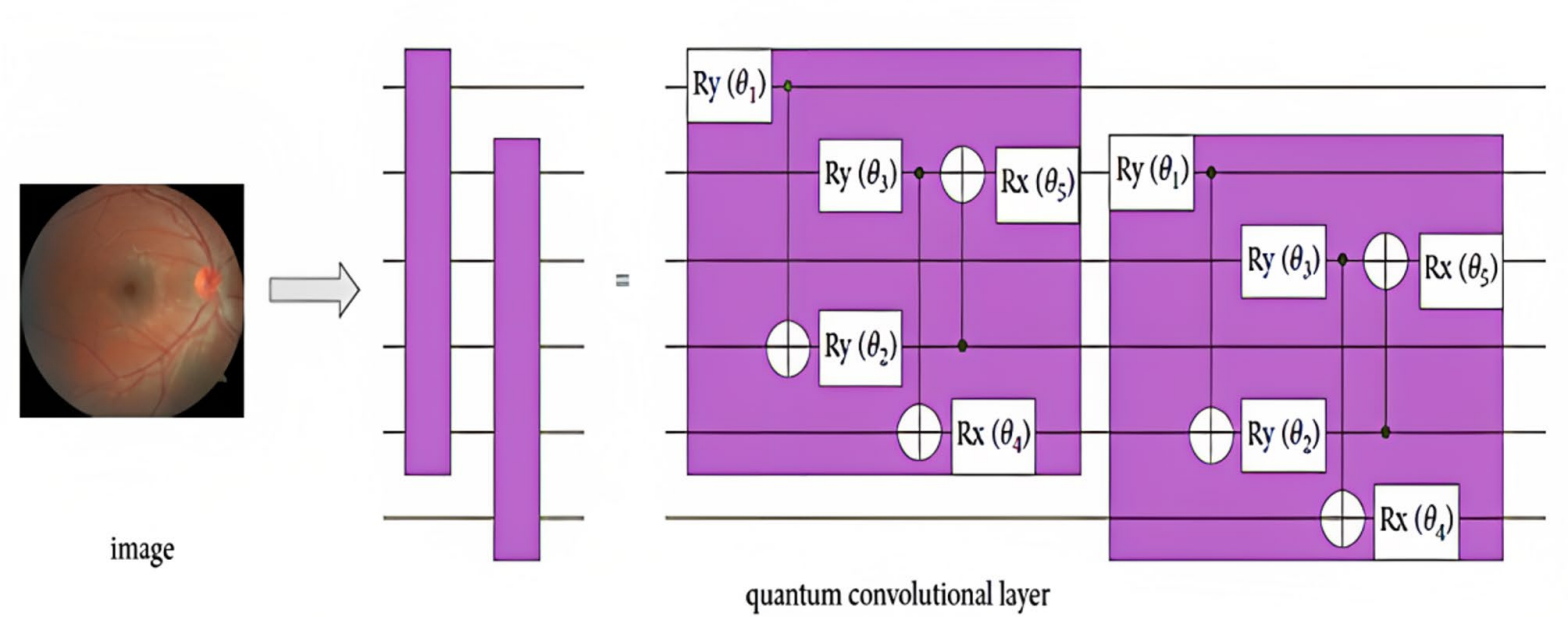
Convolutional circuit diagram.

Once the quantum state |φ_img_>, has been obtained, the PQC-designed quantum convolution kernel u(θ) is used to perform unitary conversion on |φ_img_>. (θ = θ_1_, θ_2_, θ_3_, …., θ_n_) are the learning parameters for the convolution filter. Unitary conversion is performed on the qubits of the convolution window using a convolution kernel. Quantum convolution windows equate to four qubits, but they are compatible with conventional convolution windows. By maintaining as many properties of convolution as possible while extracting hidden data from quantum states, the convolution window works on the qubits continuously. Figure 4 shows the convolutional circuit diagram. The quantum convolutional circuit diagram shows the working principle of Parameterized Quantum Circuit based kernels that operate on qubits within a convolutional window to capture hidden features in retinal images.

### Quantum pooling layer

The quantum pooling unitary gate V, which is made of three CNOT gates, is employed in this work to lower the dimensionality of the convolution findings. Convolution window output is transferred to a qubit following pooling, and the pooling window performs the identical function as convolution window output. As a result, we just evaluate the particular qubitin order to acquire the predicted result. Non - linearity is introduced into traditional CNN via nonlinear operations. We bring nonlinearity into a quantum environment by measuring it. The last quantum state |φ_out_> is acquired once the quantum environment evolves to the quantum state. To derive the anticipated value, we do Z-based assessment on the state |φ_out_>.

13 $${\rm{E}} = \left\langle {{{\rm{\varphi }}_{{\rm{img}}}}} \right|{{\rm{U}}^\dag }\left( {\rm{\theta }} \right){{\rm{V}}^\dag }\left( {{{\rm{Z}}_{1, \ldots ..,}}{{\rm{Z}}_{\rm{N}}}} \right){\rm{VU}}\left( {\rm{\theta }} \right)\left| {{{\rm{\varphi }}_{{\rm{img}}}}} \right\rangle$$

V is the constraint allowed unitary gate of the pooling layer U(θ) = u_1_(θ)u_2_(θ). . u_l_(θ), (Z_1_,. . ,Z_N_) is a vector of Z operators working on distinct qubits, l is the quantity of convolutions, in which l = (m − 1)^2^ for a picture of m × m, & the pooling unit as well accomplishes (m − 1)^2^ epochs in a pooling layer. When we openly examine the quantum convolutional layer’s result, we receive a quantum result E with size 1 × m^2^; while we analyse the quantum pooling layer’s outcome, we receive vector E with size 1 × (m − 1)^2^. Because E is a vector formed of Z expected readings of distinct qubits that is not explicitly linked to picture label, it would be sent to traditional fully connected layer for subsequent operation.

This work use learning and testing sets as assessment metrics to more naturally assess the effectiveness of HQCNN. The cross-entropy mechanism is described as below when used as the loss function:

14 $$\:\mathrm{H}=\frac{1}{\mathrm{M}}\sum\:_{\mathrm{i}}^{\mathrm{M}}{\mathrm{x}}_{\mathrm{i}}\mathrm{l}\mathrm{o}\mathrm{g}\left({\mathrm{q}}_{\mathrm{i}}\right)$$

**Fig. 5:**
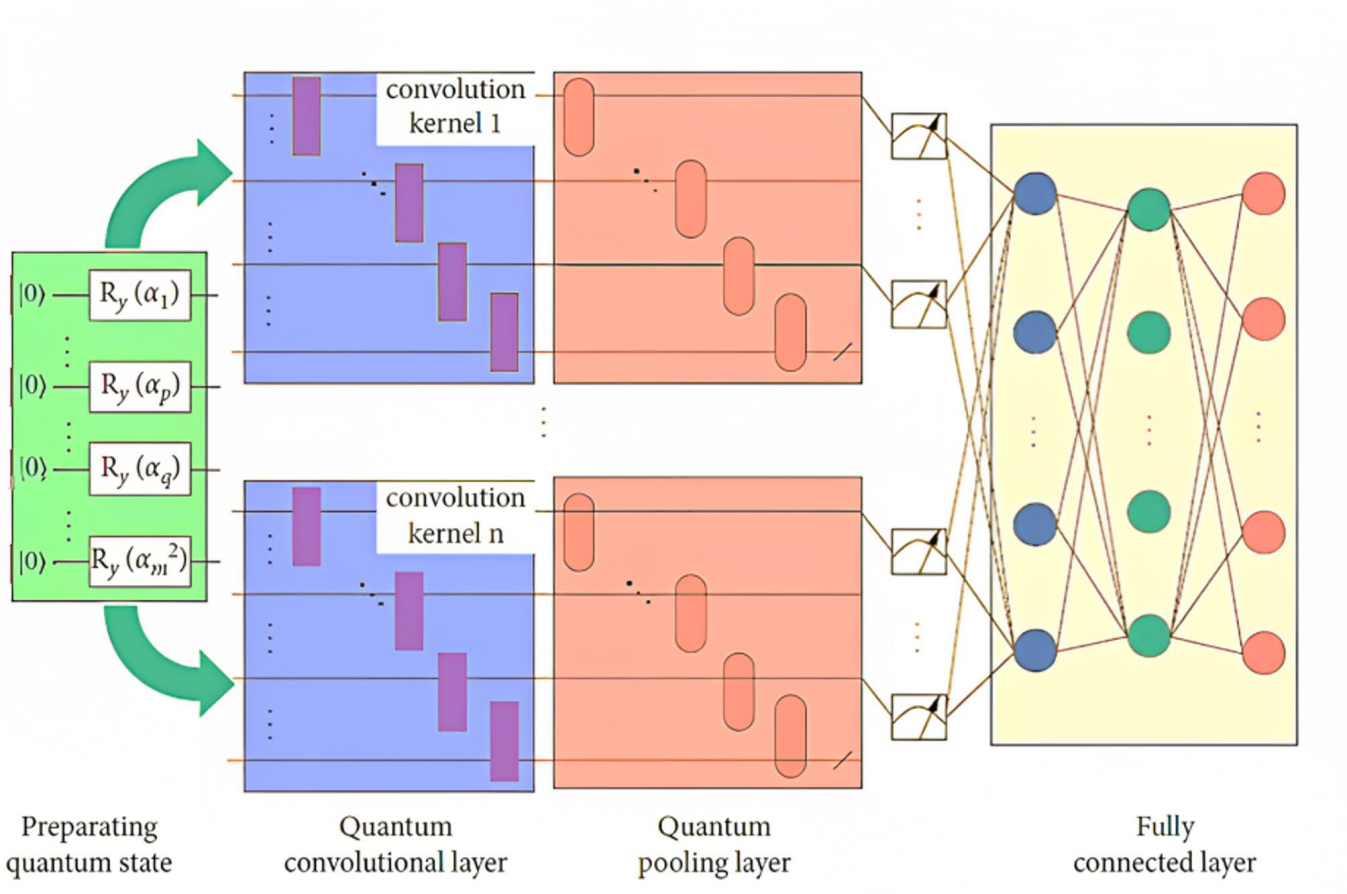
Architecture of HQCNN.

While M is the learning sample size, **x**_i_ is the one-hot vector of the ith data in the learning dataset, and q_i_ is the model’s projected likelihood vector.The architecture of HQCNN is shown in Fig. 5. The model proposed the overall architecture of Hybrid CNN. It integrates Quantum convolution and quantum pooling layers which are connected to a fully connected layer for feature extraction.

**Algorithm 1:**
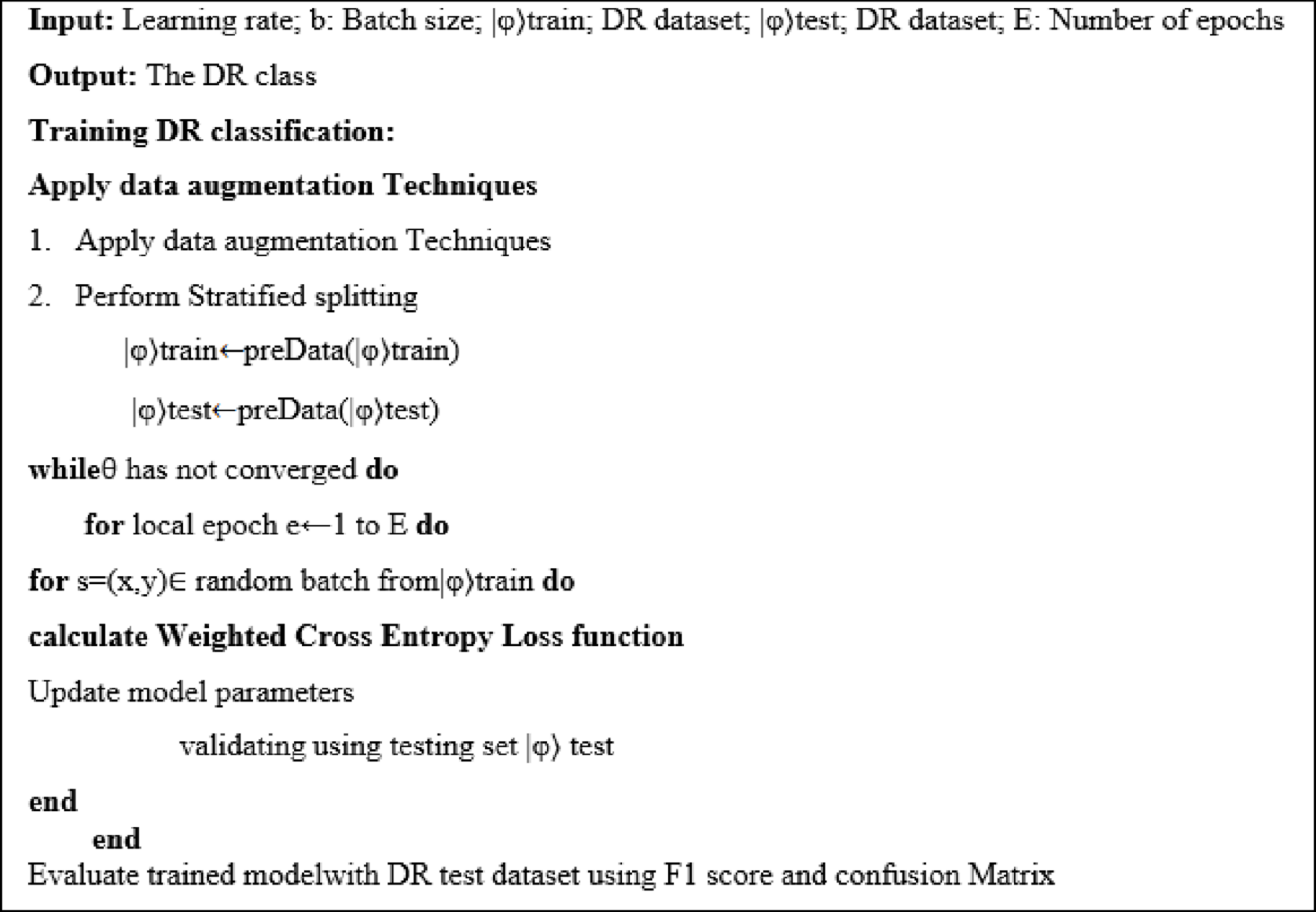
Proposed DR detection model (HQCNN).

The dataset is embedded into the HQCNN classifier. We used data transformations to increase the size of the dataset so that we could train the model. Furthermore, the number of epochs E, model parameters w, Learning Rate (LR) η, size of batch b, and the number of layers in both CNN were configured accordingly. The pseudo-code of the proposed model is given in Algorithm 1. The dataset with parameters is input into the classifier for training. For training, the data are preprocessed. |φ> train is the training data and |φ> test is the testing data. During training update, the model parameters to obtain the best results. After training the data, the model is used to test the data with the testing dataset. The results are analysed with different parameters which are listed in Table 3.

**Table 3:** Training parameters of QCNN.

Training parameters	Value(s)
Learning rate (lr)	0.001
Learning rate scheduler	Step decay with gamma = 0.5 every 5 epochs
Batch size	32
Number of epochs	50
Optimization algorithm	Adam or RMSprop
Loss function	Weighted cross entropy loss function
Quantum depth	10 (Number of quantum layers in QCNN)
Classical filters	32 (Number of classical convolutional filters)
Feature map depth	64 (Number of channels in intermediate feature maps)
Quantum gates	[RY, RZ] (Example set of quantum gates used)
Regularization	Dropout (e.g., rate = 0.5)
Data augmentation	Random rotations, flips, and translations
Evaluation metric	AUC-ROC, accuracy, sensitivity, specificity, precision, F1 score, and loss

## Results and discussion

### Computational feasibility

The proposed hybrid CNN model was implemented using PennyLane 0.17.0, Python 3.7,andPytorchand implemented on the Google Colabcloud based infrastructure with NVIDIA Tesla GPU acceleration. The average inference time per image is 13.81 ms, average training time per epoch is approximately 5–8 min and training was completed within 3–4 h.But classical CNN the average inference time per image is 30–60 ms, average training time per epoch is approximately 1.5–2 min and training was completed within 1–1.5 h. The comparative analysis of HQCNNand classical CNN shows that the higher training time per epoch due to quantum circuit simulation overhead.

The database was split by 80% for learning and 20% for assessment in the experiment. Specifically, the proposed approach is assessed utilising databases including 413, 1200, and 35,126 retinal fundus pictures from IDRiD, MESSIDOR, and KAGGLE, accordingly. IDRiD delivers 279 DR pictures and 134 regular shots, MESSIDOR offers 654 DR pictures and 546 ordinary pictures, whereas KAGGLE offers 9316 DR pictures and 25,810 typical photographs.

For multi-class classification, the IDRiD dataset includes 134 ordinary, 20 gentle NPDR, 136 modest NPDR, 74 serious NPDR, and 49 PDR photos; the MESSIDOR dataset includes 546 regular, 153 gentle DR, 247 modest DR, and 254 serious DR photos; and the KAGGLE dataset includes 25,810 common, 2443 slight NPDR, 5292 medium NPDR, 873 harsh NPDR, and 7 In binary categorization, the approach forecasts if a retinal fundus picture corresponds to DR or is healthy. Quantum K-Nearest Neighbor (QKNN), Quantum Random Forest (QRF), Quantum CNN (QCNN), Quantum Decision Tree (QDT), and HQCNN are the 4 classifiers employed in the study.

The illness prediction accuracy may be calculated as

15 $$\:\mathrm{A}\mathrm{c}\mathrm{c}\mathrm{u}\mathrm{r}\mathrm{a}\mathrm{c}\mathrm{y}=\frac{{\mathrm{T}}^{-}+{\mathrm{T}}^{+}}{{\mathrm{T}}^{-}+{\mathrm{T}}^{+}+{\mathrm{F}}^{-}+{\mathrm{F}}^{+}}$$

Here T^+^, F^+^, T−, and F^−^determine the true positive, false positive, true negative, and false negative. The sensitivity of the algorithm is then calculated as the fraction of true positives, which represents prediction when testing.

16 $$\:\mathrm{S}\mathrm{e}\mathrm{n}\mathrm{s}\mathrm{i}\mathrm{t}\mathrm{i}\mathrm{v}\mathrm{i}\mathrm{t}\mathrm{y}=\frac{{\mathrm{T}}^{+}}{{\mathrm{T}}^{+}+{\mathrm{F}}^{-}}$$

Likewise, the proportion of true negatives, which indicate the classifier’s clear identification, is used to compute specificity.

17 $$\:\mathrm{S}\mathrm{p}\mathrm{e}\mathrm{c}\mathrm{i}\mathrm{f}\mathrm{i}\mathrm{c}\mathrm{i}\mathrm{t}\mathrm{y}=\frac{{\mathrm{T}}^{-}}{{\mathrm{T}}^{-}+{\mathrm{F}}^{+}}$$

Precision measures are used to regulate the correctness and extensiveness of a classifier.

18 $$\:\mathrm{P}\mathrm{r}\mathrm{e}\mathrm{c}\mathrm{i}\mathrm{s}\mathrm{i}\mathrm{o}\mathrm{n}=\frac{{\mathrm{T}}^{+}}{{\mathrm{T}}^{+}+{\mathrm{F}}^{+}}$$

Furthermore, as illustrated below, the F1-score evaluation is critical for finding equilibrium of recall and precision.

19 $$\text{F1 score} = \frac{2\mathrm{*}(\mathrm{P}\mathrm{r}\mathrm{e}\mathrm{c}\mathrm{i}\mathrm{s}\mathrm{i}\mathrm{o}\mathrm{n}.\mathrm{R}\mathrm{e}\mathrm{c}\mathrm{a}\mathrm{l}\mathrm{l})}{(\mathrm{P}\mathrm{r}\mathrm{e}\mathrm{c}\mathrm{i}\mathrm{s}\mathrm{i}\mathrm{o}\mathrm{n}+\mathrm{R}\mathrm{e}\mathrm{c}\mathrm{a}\mathrm{l}\mathrm{l})}$$

While synthetic oversampling and cost-sensitive learning were not implemented, But the model evaluation was done using multiple Performance metrics such as sensitivity, specificity, F1 score, Precision and ROC-AUC. The high F1-score (99.8%) and sensitivity (99.8%) achieved on the Kaggle dataset show reliable detection performance for both majority and minority classes, specify efficient minority-class detection and limited majority-class dominance.

**Fig. 6:**
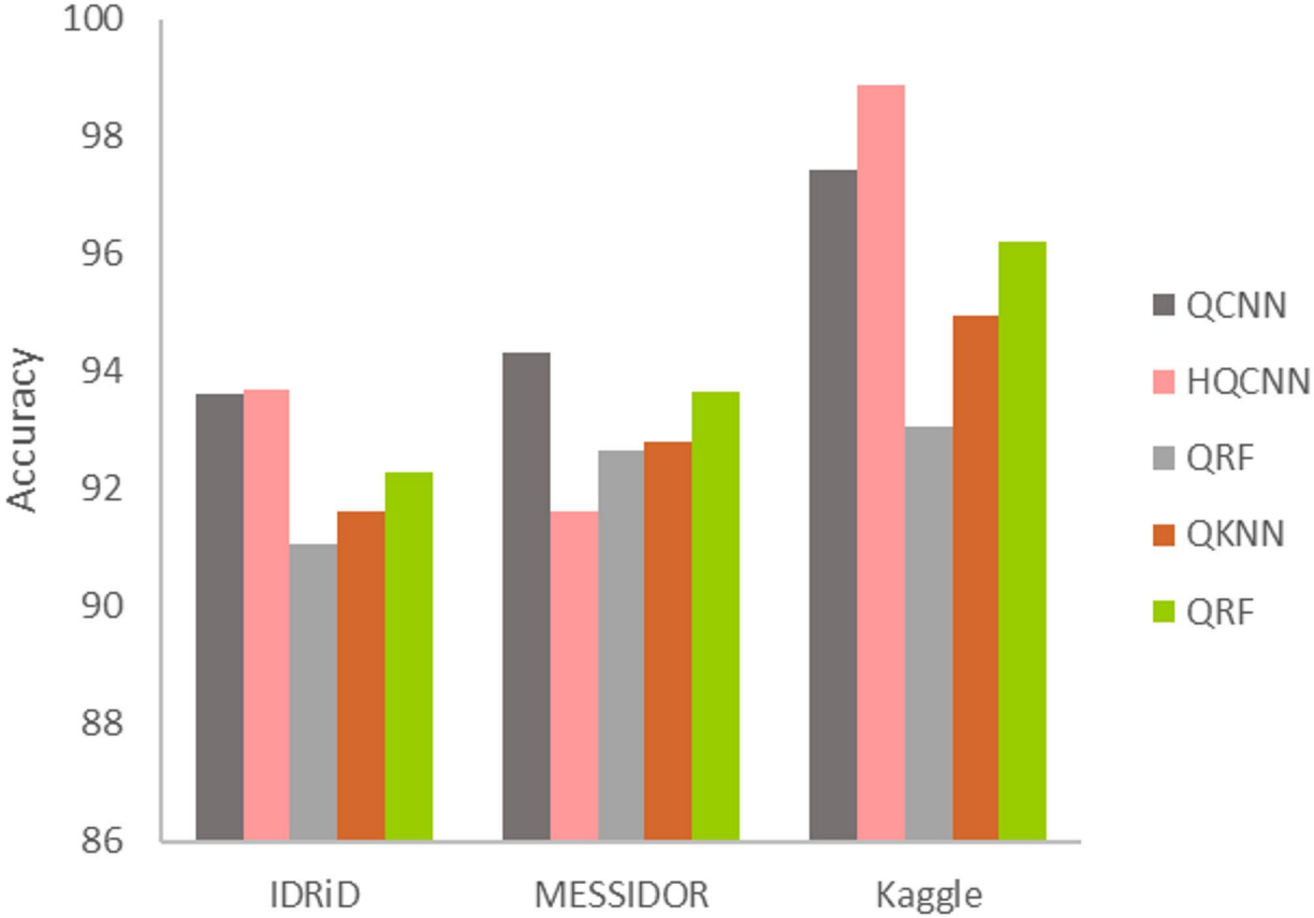
Comparative analysis of accuracy of the classifiers for different datasets.

The accuracy of the proposed and existing algorithms is shown in Fig. 6. After the investigation the projected HQCNN obtains the top accuracy of 98.89% for the Kaggle Dataset which is 5.18% higher than IDRiD dataset and 7.27% higher than MESSIDOR dataset. The QCNN obtains accuracy of 93.63% for IDRiD, 94.32% for MESSIDOR, 97.45% for Kaggle dataset. The QRF obtains accuracy of 91.07%, 92.65%, and 97.45% for IDRiD, MESSIDOR, and Kaggle dataset respectively. The QKNN obtains 91.64%, 92.83%, and 94.97% for IDRiD, MESSIDOR, and Kaggle dataset respectively. The QRF obtains 92.28%, 93.66%, and 96.21% for IDRiD, MESSIDOR, and Kaggle dataset respectively. Compared to classical models, quantum computing-based deep learning provided higher accuracy.

While the HQCNN model achieves high accuracy for Kaggle and IDRiD dataset but for MESSIDOR dataset it achieves slightly lower performance than QCNN. This behavior depends on factors such as smaller data set size and reduced variability compared to Kaggle data set.

The HQCNN model combines both classical and quantum layers which makes the model more complex one. This complex architecture requires large data sets to achieve high accuracy. The improved performance and cross data set together proves that HQCNN achieves better accuracy and scalability for large datasets.

**Fig. 7:**
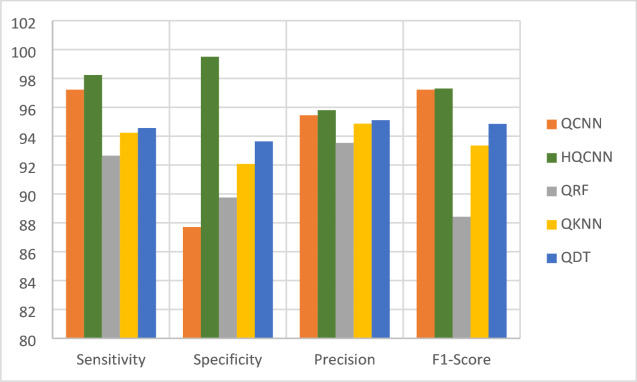
Performance comparison of the classifiers using IDRiD dataset.

The performance of the classifiers for IDRiD dataset is shown in Fig. 7. QRF obtains the sensitivity of 92.65%, specifically 89.75%, precision of 93.53%, and F1 score of 88.42%. QKNN obtains sensitivity of 94.23%, specificity of 92.08%, precision of 94.87%, and F1 score of 93.35%. QDT obtains sensitivity of 94.56%, specificity of 93.64%, precision of 95.11%, and F1 score of 94.85%. QCNN obtains the sensitivity of 97.22% of specificity of 97.71%, precision of 95.45%, and F1 score of 97.22% HQCNN obtains the sensitivity of 98.23%, specificity of 99.5%, precision of 95.8%, and F1 score of 97.3%.

**Fig. 8:**
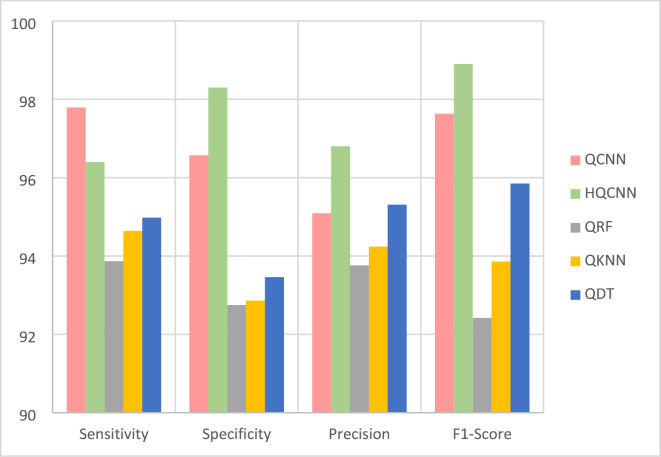
Performance comparison of the classifiers using MESSIDOR dataset.

The performance of the classifiers for MESSIDOR dataset is shown in Fig. 8. QRF obtains the sensitivity of 93.87%, specificity of 92.75%, precision of 93.76%, and F1 score of 92.42%. QKNN obtains sensitivity of 94.64%, specificity of 92.86%, precision of 94.24%, and F1 score of 93.86%. QDT obtains sensitivity of 94.98%, specificity of 93.46%, precision of 95.31%, and F1 score of 95.85%. QCNN obtains the sensitivity of 97.79% of specificity of 93.57%, precision of 95.09%, and F1 score of 97.63% HQCNN obtains the sensitivity of 96.40%, specificity of 98.32%, precision of 96.85%, and F1 score of 98.93%.

**Fig. 9:**
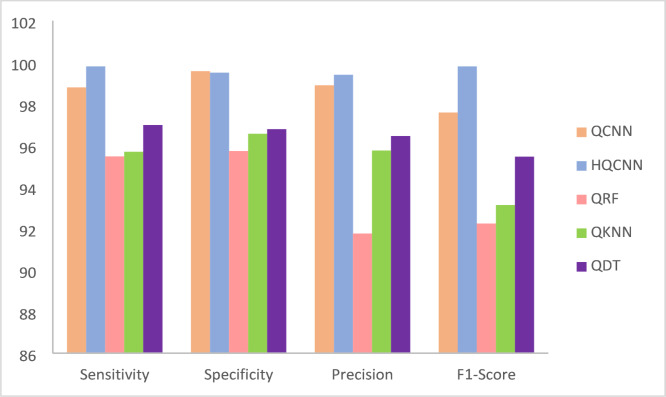
Performance comparison of the classifiers using Kaggle dataset.

The performance of the classifiers for Kaggle dataset is shown in Fig. 9. QRF obtains sensitivity of 95.47%, specificity of 95.73%, precision of 91.76%, and F1 score of 92.24%. QKNN obtains the sensitivity of 95.69%, specificity of 96.56%, precision of 95.75%, and F1 score 93.13%. QDT obtains the sensitivity of 96.98%, specifically of 96.78%, precision of 96.45%, and F1 score of 95.46%. QCNN obtains the sensitivity of 98.79% of specificity of 99.57%, precision of 98.89%, and F1 score of 97.58% HQCNN obtains the sensitivity of 99.8%, specificity of 99.5%, precision of 99.4%, and F1 score of 99.8%.

### Receiver operator characteristic (ROC) curve

**Fig. 10:**
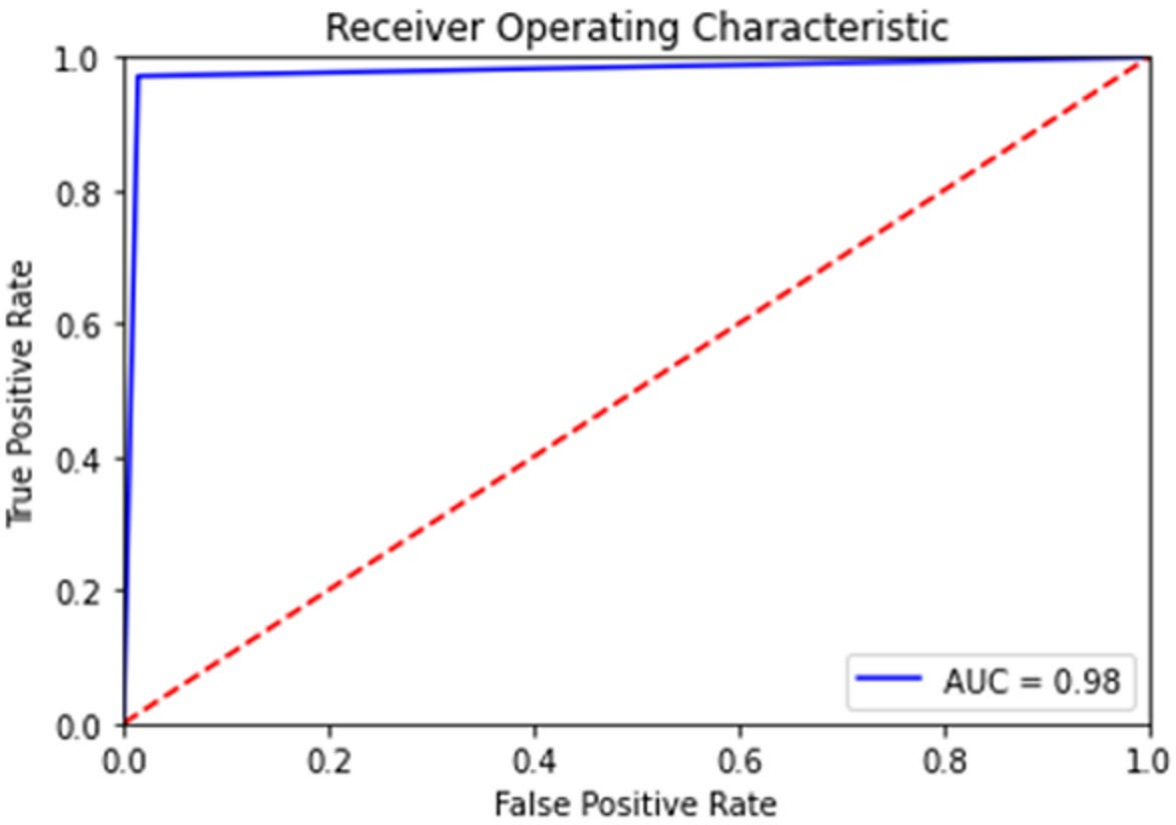
ROC between normal retinal image and DR.

A ROC curve appears to be required for problem identification then categorization. This ROC is a probability curve that compares the True Positive Rate (TPR) to the False Positive Rate (FPR) at several threshold settings to distinguish the signal from background noise. TPR, or sensitivity, is a metric that measures how well the negative category is evaluated. The FPR, also known as specificity, indicates how much of the negative group the model incorrectly classifies. The AUC metric quantifies the ROC curve, which computes a model’s ability to discriminate among classes. The ROC curve in the top left corner demonstrates that the proposed study correctly identifies DR. The ROC graph is presented in Fig. 10, corresponds to binary classification model. In this class (1–4) refers to the severity in DR classes together called as positive class and Class 0 represents the negative class.

**Fig. 11:**
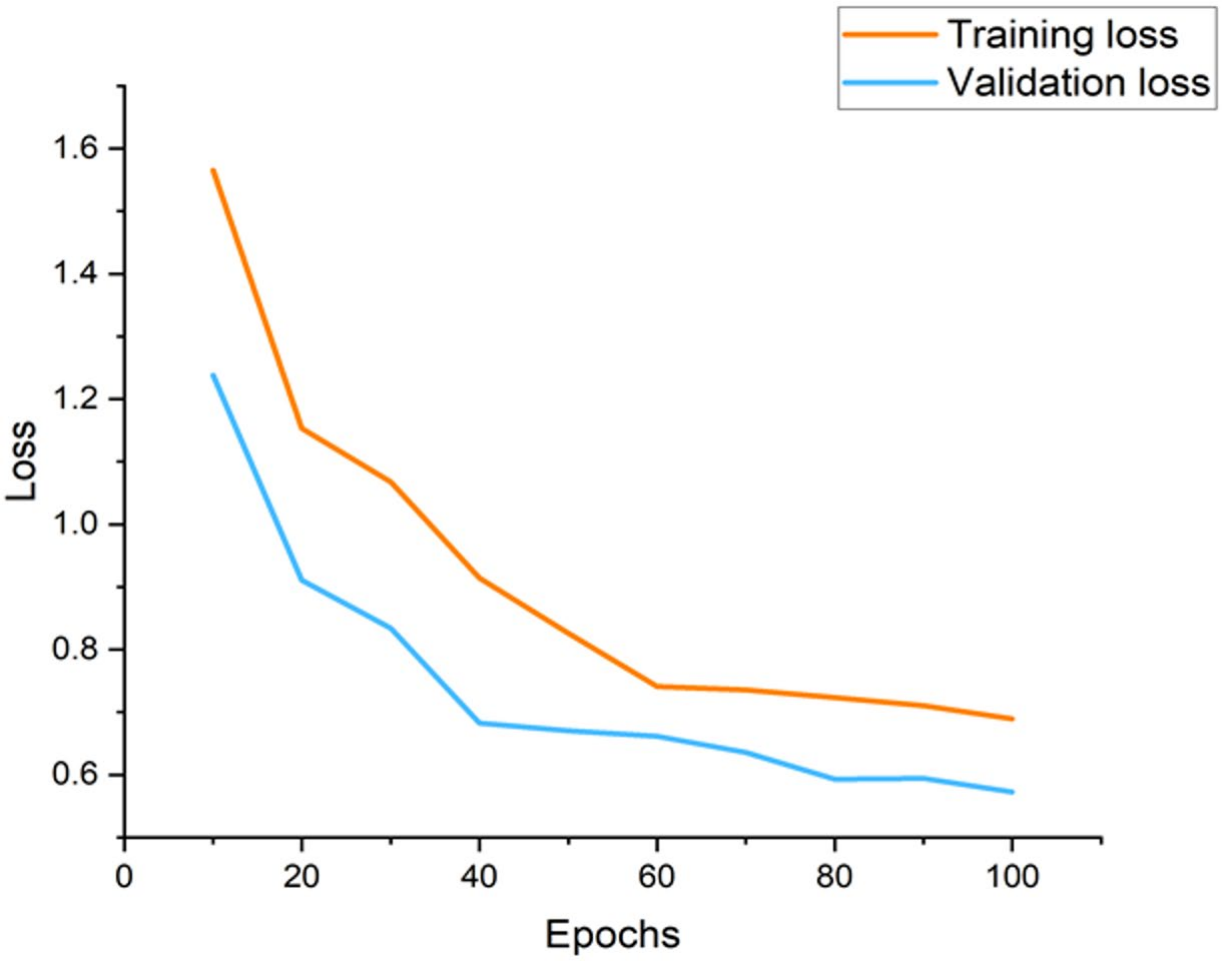
Loss of proposed HQCNN.

**Fig. 12:**
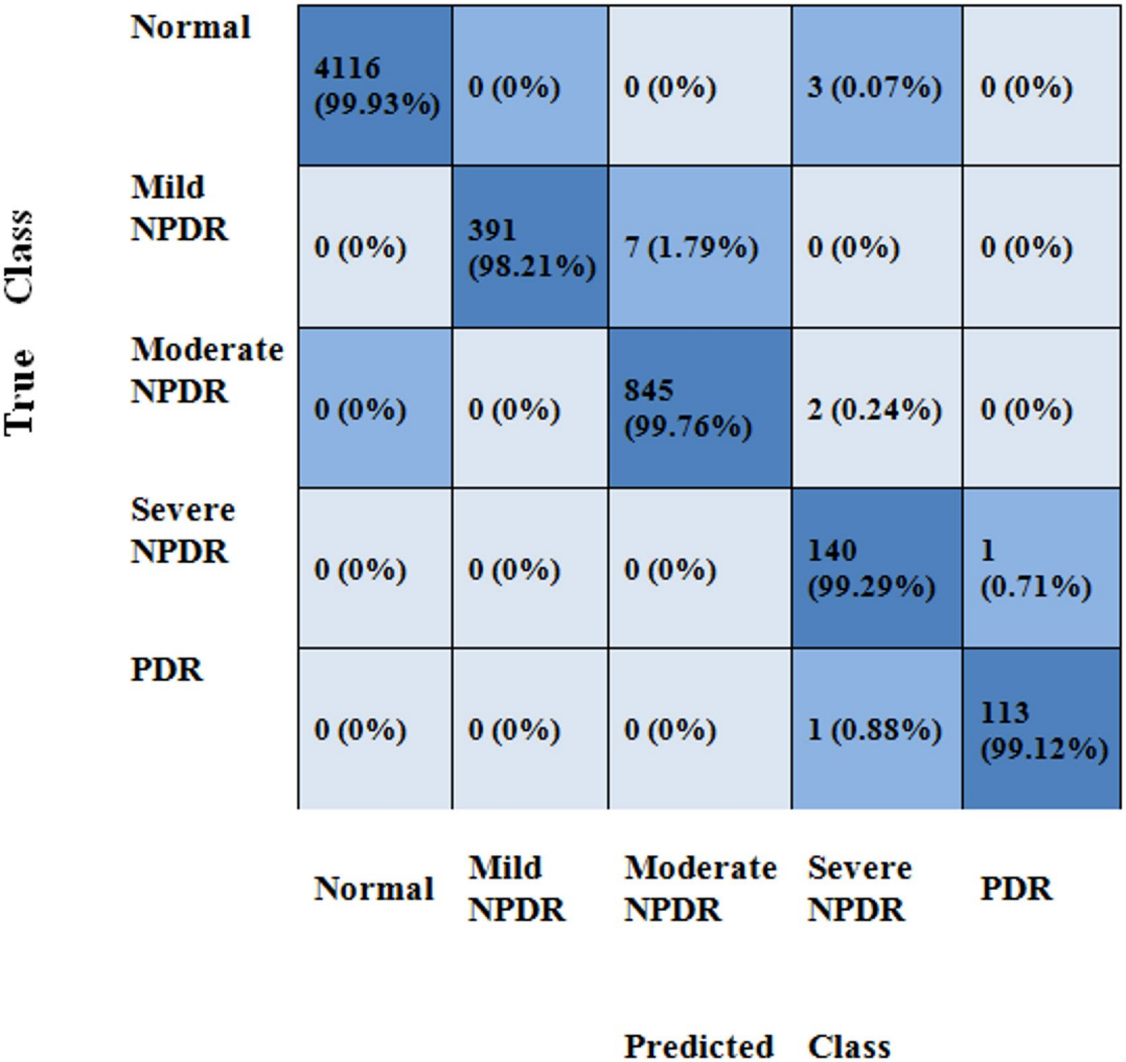
Confusion matrix for assessing proposed HQCNN classifiers for multi-class classification using the KAGGLE database.

### Training and validation loss

Among the most significant elements of NN is the loss function, which is a forecast error of the framework. Figure 11 displays the model loss for HQCNN throughout training and validation. The design loss for epochs = 100 is depicted in this graph. By changing the weight vector ratios and employing the Adam optimizing strategy, the worth of the loss functional is lowered in respect to the framework’s properties in this work. In comparison to previous models, the proposed approach has a lower validation loss because of excellent training using a large dataset. They are trained to cope with complex issues in difficult imaging environments. It could be capable of leaving the study atmosphere and becoming functional tools because of their tenacity. The confusion matrix for the proposed work is presented in Fig. 12. Regarding performance, this method surpasses existing classification approaches. The observed outcomes show that the anticipated framework beats the Kaggle database in categorization trials. When contrasted to other methodologies, the efficiency of the HQCNN for the Kaggle database is 98.9%. The performance of quantum computing is better because the Quantum computers use quantum bits, or qubits, to measure and extract information. Unlike the bits of classical computers, which can store a 1 or 0, qubits can store multiple values at the same time. This gives them a huge speed advantage over classical computers and algorithms. Deep learning models made on quantum computers are more powerful and faster compared to the models that are run on classical computers. Quantum deep learning helps to solve problems with complex correlations between inputs that are challenging to solve with traditional classic computers. The quantum convolution layer extracts the most important features from the dataset, which is most important for the detection of diseases. By training the learning algorithm with most important features, the performance of the algorithm can be improved (Table 4).

### Comparison with classical models

**Table 4:** Comparative analysis of HQCNN with SOTA classical CNN.

Model	Accuracy (%)	Sensitivity (%)	Specificity (%)	Precision (%)	F1 Score(%)
HQCNN	98.89	98.79	99.57	98.89	97.58
ResNet -50	97.65	96.80	98.20	96.90	96.85
EfficientNet -80	97.90	97.10	98.30	97.20	97.15
VGG16	96.85	95.70	97.50	95.80	95.75

**Fig. 13:**
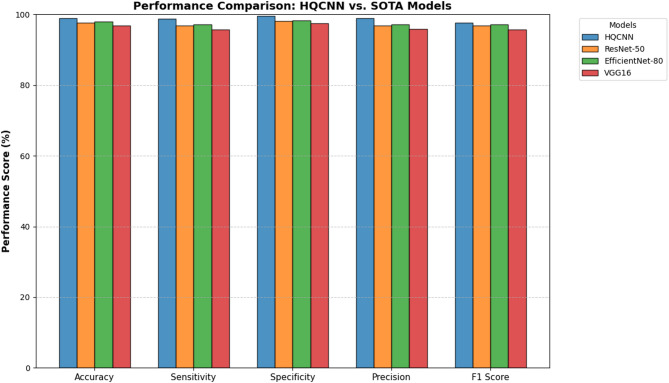
Comparative performance analysis of HQCNN for binary classification with other SOTA classical CNN models using KAGGLE d atabase.

**Fig. 14:**
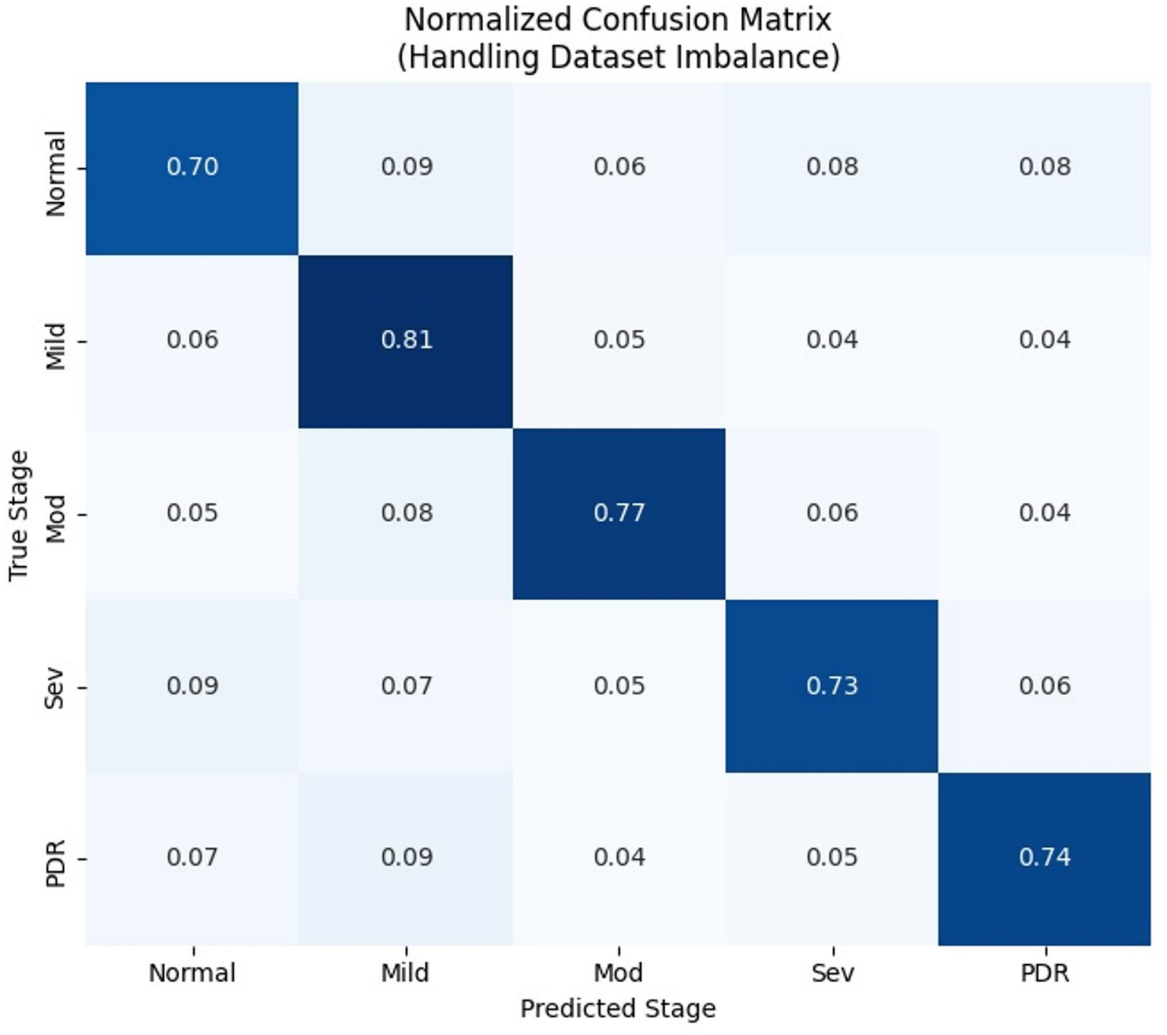
Confusion matrix for Data Imbalance using Kaggle dataset.

The performance of the classifiers for Kaggle dataset is done for classical CNN methods. The HQCNN achieves better performance in accuracy, sensitivity, specificity, precision and F1 score compared to classical CNN such as ResNet- 50, EfficientNet – 80 and VGG16.It uses Binary classification task for achieving above 95%of accuracy (Figs. 13, 14).

### Dataset imbalance confusion matrixes

The normalized confusion matrix is calculated for Kaggle dataset using weighted cross entropy loss function. So that it obtained an average of 75% F1-score.

**Fig. 15:**
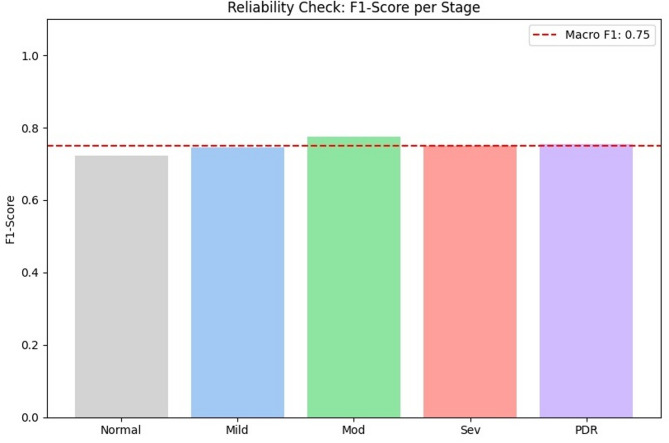
Performance analysis of F1 score across imbalanced classes.

The performance analysis due to the nature of the classification tasks. The classical CNN versus HQCNN comparison in Fig. 13 show the model’s ability to perform Binary classification of normal versus Disease DR, which is a critical screening metric. Conversely, the F1-score of 0.75 in Fig. 15 represents the Severity Grading of 5-classes. This distinction is crucial, as grading involves distinguishing between subtle pathological features in adjacent stages (e.g., Mild vs. Moderate), which presents a higher objective difficulty. The model maintains reliability over the Moderate and Severe stages. It ensures the clinical safety.

### Computational efficiency

**Table 5:** Computational efficiency of HQCNN with SOTA classical CNN.

Model	Total parameters (M)	Model size (MB)	Inference time (ms)
VGG 16	138.40	528.0	18.20
ResNet-50	25.60	98.0	7.40
EfficientNet-B0	5.30	20.0	4.10
Proposed HQCNN	1.00	4.2	13.81

The computational efficiency of Hybrid Quantum Convolutional Neural Network is shown in above Table 5, HQCNN utilized only 1 million parameters taken from the model size of 4.2 MB with inference time of 13.81ms. HQCNN model represents 96% reduction in model size compared to ResNet-50 and 99% reduction in VGG 16. It’s computed using Google Colab of Pytorch, Telsa T4 GPU,12 GB RAM.

## Conclusion

This work proposed a fusion quantum convolutional computing paradigm in this paper. A quantum convolutional layer and a quantum pooling layer are included in the quantum computing component. To execute the linear unitary conversion of the quantum state, the quantum convolutional layer adopts a parameterized quantum circuit strategy. Using parameterized quantum circuits, the power of quantum computing can be realized. This enhanced the outcomes by categorizing the multi-class categorization of DR in the experimental simulation using the Kaggle database. The findings show that, when compared to other quantum-based algorithms, HQCNN has quicker and more efficient training time. In the future, quantum computing could be used to design supersonic drugs, conduct in silico clinical trials with virtual humans, sequence and analyze whole genomes at full speed, migrate hospitals to the cloud, develop predictive health, or secure medical data using quantum uncertainty. Medical images can be analyzed more efficiently using quantum computing, including edge detection and image matching. Diagnostics using image-aided technology would be significantly improved with these improvements. Future work will concentrate on clinical integration in ophthalmology departments to evaluate patient outcomes.

## Data Availability

The data is available from the corresponding author upon reasonable request.
